# Development and validation of a novel risk score for predicting post-ERCP pancreatitis in patients with choledocholithiasis: a retrospective study

**DOI:** 10.1186/s12876-026-04708-6

**Published:** 2026-03-25

**Authors:** Peng Cong, Zhen Liu, Min Wei

**Affiliations:** Department of Gastroenterology, Liuzhou People’s Hospital, Liuzhou, Guangxi 545000 China

**Keywords:** Post-ERCP pancreatitis, Choledocholithiasis, Risk prediction model, Logistic regression, Bootstrap validation

## Abstract

**Introduction:**

Post-endoscopic retrograde cholangiopancreatography (ERCP) pancreatitis (PEP) remains the most common serious complication of ERCP, with variable incidence across patient populations. Choledocholithiasis patients represent a distinct subgroup with unique risk profiles, yet existing prediction models lack indication-specific validation. We aimed to develop and internally validate a practical risk score for predicting PEP in patients undergoing ERCP for choledocholithiasis.

**Methodology:**

This single-center retrospective cohort study included 500 consecutive patients who underwent therapeutic ERCP for choledocholithiasis at Liuzhou People’s Hospital between January 2019 and December 2024. PEP was defined according to Cotton consensus criteria. Candidate predictors were selected based on literature review and clinical availability. Multivariable logistic regression with least absolute shrinkage and selection operator (LASSO) regularization was employed for variable selection. The final model was internally validated using 500 bootstrap iterations with optimism correction. Model performance was assessed through discrimination (C-statistic), calibration (calibration slope, Hosmer-Lemeshow test), and overall fit (Brier score). A simplified integer-based risk score was developed for clinical application.

**Results:**

PEP occurred in 76 patients (15.20%), with mild, moderate, and severe cases representing 72.37%, 23.68%, and 3.95% of events, respectively. Five independent predictors were retained in the final model: prior post-ERCP pancreatitis (adjusted odds ratio [aOR] 4.38, 95% confidence interval [CI] 1.92–10.03), asymptomatic choledocholithiasis presentation (aOR 6.57, 95% CI 3.52–12.25), difficult cannulation (aOR 2.50, 95% CI 1.43–4.39), pancreatic duct instrumentation (aOR 1.93, 95% CI 1.12–3.33), and precut sphincterotomy (aOR 3.43, 95% CI 1.66–7.08). The model demonstrated acceptable discrimination with an optimism-corrected C-statistic of 0.760 (95% CI 0.708–0.817) after bootstrap internal validation. Calibration was satisfactory (Hosmer-Lemeshow *p* = 0.967; calibration slope 0.939). The derived risk score stratified patients into low (0–1 points; 6.92% PEP rate), moderate (2–3 points; 21.85% PEP rate), and high-risk (≥ 4 points; 44.44% PEP rate) categories.

**Conclusions:**

We developed and internally validated a parsimonious, choledocholithiasis-specific risk score demonstrating robust discrimination and calibration for PEP prediction. This practical tool enables individualized risk stratification in patients undergoing ERCP for common bile duct stones. The clinical utility of risk-stratified prophylaxis requires evaluation through external validation studies and prospective trials assessing patient outcomes before clinical implementation can be recommended.

## Introduction

Endoscopic retrograde cholangiopancreatography (ERCP) has become the cornerstone therapeutic modality for managing biliary tract disorders, particularly choledocholithiasis [1]. Despite significant technical advances and increasing operator expertise over the past four decades, post-ERCP pancreatitis (PEP) remains the most frequent serious complication, accounting for substantial morbidity, mortality, and healthcare costs [2, 3]. Contemporary meta-analyses report overall PEP incidence of approximately 10%, with rates substantially higher in high-risk patient subgroups reaching 14–15% [2]. 

Choledocholithiasis represents one of the most common indications for therapeutic ERCP, yet patients with common bile duct (CBD) stones demonstrate heterogeneous risk profiles depending on clinical presentation and stone characteristics. Recent evidence suggests that asymptomatic choledocholithiasis confers markedly elevated PEP risk compared to symptomatic presentations, with pooled odds ratios approaching 4.0-4.8 [3, 4]. This paradoxical finding—wherein seemingly “less sick” patients face higher complication rates—underscores the critical need for robust, indication-specific risk stratification tools to guide prophylactic strategies.

Over the past two decades, numerous PEP prediction models have been developed, incorporating patient-related factors (prior pancreatitis, female sex, younger age), disease-related factors (sphincter of Oddi dysfunction, suspected pancreatic disease), and procedure-related factors (difficult cannulation, pancreatic duct instrumentation, precut sphincterotomy) [5–7]. A recent systematic review identified [8] published prediction models encompassing 38,016 ERCP procedures, yet only 46% underwent external validation, and methodological quality concerns were pervasive [9]. Critically, the vast majority of existing models were developed in general ERCP populations, with limited attention to indication-specific risk profiles. Among choledocholithiasis patients specifically, validated prediction tools remain scarce despite this being the predominant ERCP indication in most centers [10–12]. 

Current American Society for Gastrointestinal Endoscopy (ASGE) guidelines recommend risk-stratified prophylaxis, endorsing rectal nonsteroidal anti-inflammatory drugs (NSAIDs) for all patients and prophylactic pancreatic stent placement for high-risk cases [13]. However, practical implementation of these recommendations is hampered by the absence of widely accepted, easy-to-use risk calculators that can be applied at the point of care. Furthermore, recent trials have demonstrated that combination prophylaxis may not be superior to NSAIDs alone in certain contexts, emphasizing the importance of precise risk estimation to optimize resource allocation and minimize unnecessary interventions [14, 15]. 

To address these gaps, we aimed to develop and internally validate a novel, practical risk score specifically for predicting PEP in patients undergoing therapeutic ERCP for choledocholithiasis. We employed contemporary methodological standards for prediction model development, including LASSO-based variable selection, bootstrap internal validation with optimism correction, and comprehensive performance assessment encompassing both discrimination and calibration. The resulting tool is designed for immediate clinical application, facilitating individualized risk counseling and guiding prophylactic intervention selection in this high-volume patient population.

## Methods

### Study design and setting

This single-center retrospective cohort study was conducted at Liuzhou People’s Hospital, a tertiary referral center in Guangxi Province, China, serving a catchment population of approximately 4 million. The study was designed and reported according to the Transparent Reporting of a multivariable prediction model for Individual Prognosis or Diagnosis (TRIPOD) statement [16, 17] and Strengthening the Reporting of Observational Studies in Epidemiology (STROBE) guidelines [18]. The institutional review board of Liuzhou People’s Hospital approved this study (Approval number: KY2025-173-01) with waiver of informed consent due to the retrospective nature and use of de-identified data.

### Study population

We included all consecutive adult patients (age ≥ 18 years) who underwent therapeutic ERCP with intended bile duct stone extraction at our institution between January 1, 2019, and December 31, 2024. Choledocholithiasis was confirmed by pre-procedure imaging (magnetic resonance cholangiopancreatography [MRCP], computed tomography [CT], or transabdominal ultrasound) and endoscopic findings. Exclusion criteria were: (1) prior biliary sphincterotomy; (2) suspected or confirmed chronic pancreatitis; (3) concurrent acute pancreatitis at the time of ERCP; (4) malignant biliary obstruction; (5) ERCP performed for indications other than choledocholithiasis; (6) incomplete procedural documentation precluding outcome ascertainment; and (7) pregnancy.

### Outcome definition

The primary outcome was PEP, defined according to Cotton consensus criteria as new or worsened abdominal pain persisting for at least 24 h after the procedure, serum amylase elevation to at least three times the upper limit of normal at 24 h post-procedure, and requirement for hospitalization or prolongation of planned admission for at least 2 days [19]. PEP severity was classified as mild (2–3 days hospitalization), moderate (4–10 days hospitalization), or severe (> 10 days hospitalization, hemorrhagic pancreatitis, intensive care unit admission, or need for percutaneous drainage or surgery) [19–21]. 

Serum amylase and lipase were routinely measured at 4–6 h and 24 h post-procedure as per institutional protocol. Clinical outcomes were ascertained through systematic review of electronic medical records, including physician notes, laboratory results, imaging reports, and discharge summaries. Two independent investigators (CP and ZL) performed data abstraction using standardized case report forms, with discrepancies resolved through consensus discussion.

#### Candidate predictors

Candidate predictors were selected a priori based on comprehensive literature review of established PEP risk factors and clinical availability in routine practice [5, 7, 9]. We focused on variables that could be reliably extracted from electronic medical records and were measured or documented prospectively during clinical care. Candidate predictors were categorized as follows:

##### Patient-related factors

Age, sex, body mass index (BMI), prior acute pancreatitis history, prior post-ERCP pancreatitis, chronic pancreatitis.

##### Clinical presentation

Categorized as cholangitis, symptomatic choledocholithiasis (biliary colic without cholangitis), or asymptomatic choledocholithiasis (incidentally discovered stones).

##### Laboratory parameters (pre-procedure)

Total bilirubin, alanine aminotransferase (ALT), aspartate aminotransferase (AST), alkaline phosphatase (ALP), gamma-glutamyl transferase (GGT), baseline serum amylase and lipase, white blood cell count, C-reactive protein.

##### Imaging characteristics

CBD diameter (dilated defined as > 8 mm for patients without cholecystectomy, > 10 mm post-cholecystectomy), stone size (categorized as < 5 mm, 5–10 mm, 10–15 mm, > 15 mm), number of stones (multiple stones defined as > 3), intrahepatic duct dilation.

##### Procedure-related factors

Native papilla status, difficult cannulation (defined as > 5 min or > 5 attempts to achieve deep biliary cannulation), unintentional pancreatic duct cannulation, pancreatic duct wire passage, pancreatic contrast injection, sphincterotomy type (standard vs. precut), pancreatic sphincterotomy, balloon dilation of intact papilla, stone extraction method, prophylactic pancreatic stent placement.

All continuous variables were initially considered in their original scale to maximize information content, with subsequent categorization based on clinical relevance and inspection of functional relationships.

### Statistical analysis

#### Descriptive statistics

Baseline characteristics were summarized and compared between patients with and without PEP. Continuous variables were assessed for normality using the Shapiro-Wilk test. Normally distributed variables were expressed as mean ± standard deviation and compared using Student’s t-test; non-normally distributed variables were expressed as median (interquartile range) and compared using the Mann-Whitney U test. Categorical variables were expressed as frequencies (percentages) and compared using the chi-square test or Fisher’s exact test as appropriate. Statistical significance was defined as two-sided *p* < 0.05.

#### Sample size justification

Sample size was determined using contemporary criteria for prediction model development proposed by Riley et al. [22, 23] For a binary outcome with anticipated event fraction of 0.10, target C-statistic of 0.75, maximum of 5 candidate predictors, and desired shrinkage factor ≥ 0.90, the required sample size was calculated as approximately 450–500 participants. Our cohort of 500 patients with 76 PEP events (events per variable [EPV] = 15.2) satisfied these criteria and exceeded the traditional rule of EPV ≥ 10.

#### Variable selection and model development

We employed least absolute shrinkage and selection operator (LASSO) logistic regression with 10-fold cross-validation to perform embedded variable selection from the candidate predictor set [24]. LASSO applies L1 penalty to shrink less important predictor coefficients toward zero, effectively performing automatic variable selection while reducing overfitting risk. The optimal penalty parameter (λ) was selected based on maximum area under the receiver operating characteristic curve (AUC) in cross-validation.

Following LASSO variable selection, we applied pre-specified reduction criteria to identify the final parsimonious model: (1) statistical significance threshold of *p* < 0.05 in both univariable analysis and initial multivariable model; (2) effect size magnitude with adjusted odds ratio ≥ 1.5 or ≤ 0.67; (3) clinical actionability, prioritizing modifiable procedural factors and established risk factors with biological plausibility; and (4) sample size constraint requiring events per variable (EPV) ≥ 10 to minimize overfitting risk.

Following LASSO selection, a final multivariable logistic regression model was fit without penalization to obtain unbiased coefficient estimates, standard errors, and odds ratios. Model assumptions were verified through assessment of linearity (for continuous predictors), multicollinearity (variance inflation factor < 5), and influential observations (Cook’s distance). The full model equation with intercept and all coefficients was reported to facilitate external validation.

#### Internal validation

Internal validation was performed using Harrell’s bootstrap optimism correction procedure with 500 bootstrap iterations [8, 25, 26]. For each bootstrap iteration, we: (1) drew a bootstrap sample with replacement from the original dataset (*n* = 500); (2) developed the full modeling procedure (identical variable selection and estimation procedures) in the bootstrap sample; (3) calculated performance measures in both the bootstrap sample (training performance) and the original sample (test performance); and (4) computed optimism as the difference between bootstrap training and test performance. The average optimism across 500 iterations was subtracted from the apparent (naive) performance to obtain optimism-corrected estimates.

#### Model performance assessment

Model performance was evaluated across three dimensions: discrimination, calibration, and overall fit [27]. 

*Discrimination* was quantified using the C-statistic (equivalent to area under the ROC curve for binary outcomes), which represents the probability that a randomly selected patient with PEP has a higher predicted probability than a randomly selected patient without PEP. C-statistic values of 0.70–0.80 indicate acceptable discrimination, while > 0.80 indicates excellent discrimination [28]. We calculated 95% confidence intervals using the bootstrap distribution of optimism-corrected C-statistics. The optimal probability threshold was determined using Youden’s J statistic (sensitivity + specificity − 1), and corresponding sensitivity, specificity, positive predictive value (PPV), and negative predictive value (NPV) were reported.

*Calibration* was assessed through multiple complementary approaches: [29, 30] (1) calibration-in-the-large, comparing mean predicted probability to observed event rate (ideal = 0); (2) calibration slope, derived from a logistic model regressing observed outcomes on logit-transformed predicted probabilities (ideal = 1.0); (3) calibration plot, displaying observed versus predicted event rates across deciles of predicted probability with LOESS smoothing; and (4) Hosmer-Lemeshow goodness-of-fit test, evaluating agreement between observed and expected events across risk deciles (*p* > 0.05 indicates adequate fit) [31, 32]. 

*Overall performance* was quantified using the Brier score, calculated as the mean squared difference between predicted probabilities and observed binary outcomes (range 0–1, lower is better). The Brier score simultaneously captures aspects of both discrimination and calibration [27, 33]. 

Both apparent (naive) performance and bootstrap optimism-corrected performance were reported for all metrics.

#### Risk score development

To facilitate clinical implementation, we transformed the final regression model into a simplified integer-based risk score following established methodology [34, 35]. Regression coefficients were divided by the smallest non-zero coefficient and rounded to the nearest integer to generate point values for each predictor. Total risk scores were calculated as the sum of points across all present predictors. We then categorized patients into risk groups (low, moderate, high) based on observed PEP rates, ensuring clinically meaningful separation between groups and practical utility for decision-making.

#### Missing data

The extent and patterns of missing data were characterized for all variables. Variables with > 20% missingness were excluded from consideration as candidate predictors. For variables with < 20% missingness, we examined whether missingness was associated with outcome status (missing at random vs. missing not at random). Given the retrospective nature and focus on routinely collected clinical variables, missing data rates were generally low (< 5%), and complete-case analysis was employed. Sensitivity analyses using multiple imputation by chained equations were conducted for key results.

#### Software

All statistical analyses were performed using Python version 3.12 (Python Software Foundation) with scikit-learn (version 1.3.2) for LASSO regression and model development, pandas (version 2.1.4) for data manipulation, and matplotlib/seaborn (versions 3.8.2/0.13.0) for visualization. Statistical significance was set at two-sided α = 0.05 for all tests.

## Results

### Study population and baseline characteristics

**Fig. 1:**
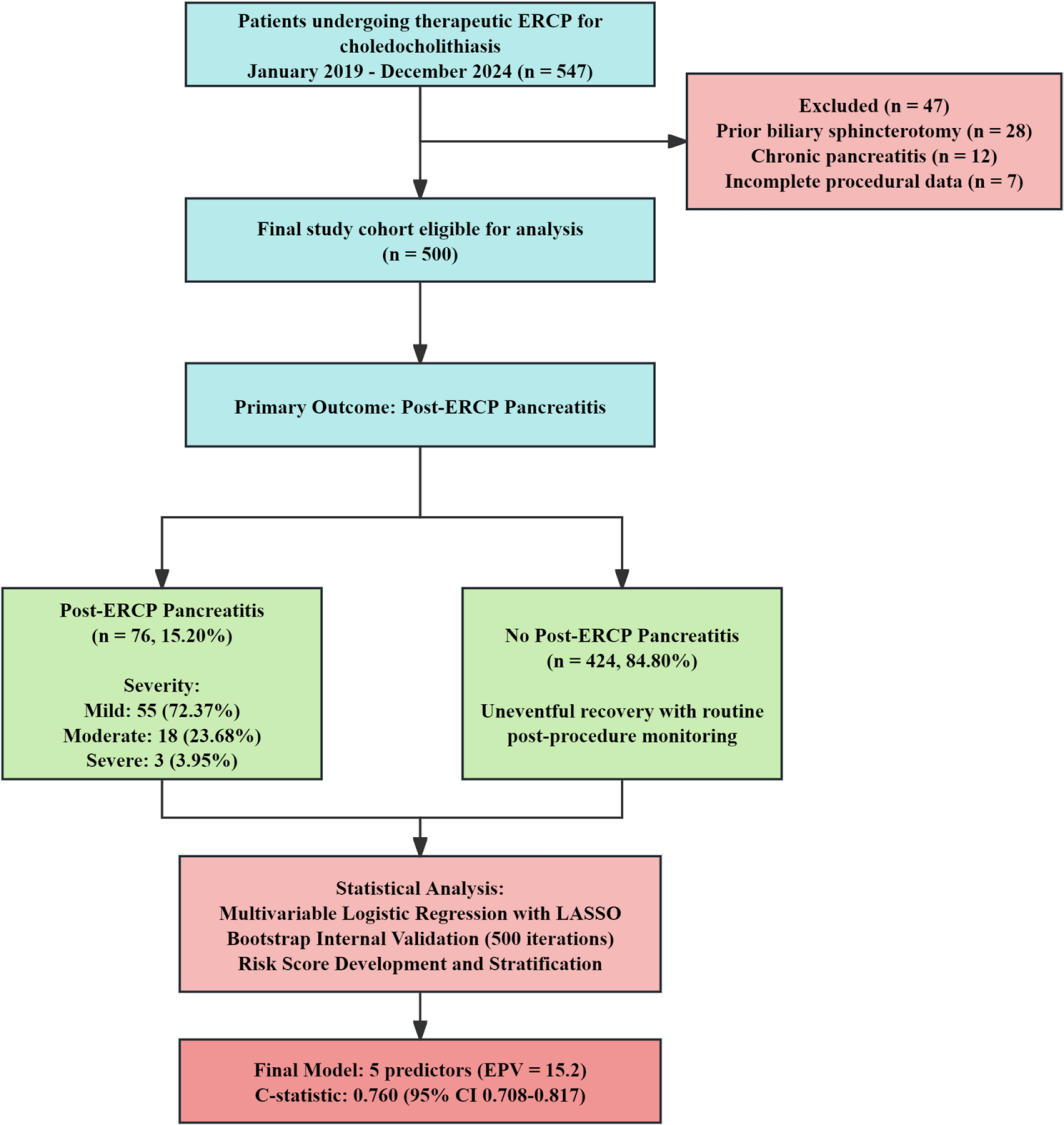
Study flow diagram

Between January 2019 and December 2024, 547 patients underwent therapeutic ERCP for choledocholithiasis at our institution. After applying exclusion criteria (prior sphincterotomy, *n* = 28; chronic pancreatitis, *n* = 12; incomplete data, *n* = 7), 500 patients were included in the final analysis (Fig. 1). The median age was 62 years (range 25–90), and 285 patients (57.00%) were female. The majority of patients presented with symptomatic disease: cholangitis (*n* = 224, 44.80%), biliary colic (*n* = 209, 41.80%), or asymptomatic choledocholithiasis (*n* = 67, 13.40%).

Post-ERCP pancreatitis occurred in 76 patients, representing an overall incidence of 15.20% (95% CI 12.15–18.65%). Among PEP cases, severity distribution was: mild 55 cases (72.37%), moderate 18 cases (23.68%), and severe 3 cases (3.95%). No PEP-related deaths occurred during the study period. Median length of hospital stay was significantly longer for PEP patients (5 days, IQR 3–7) compared to non-PEP patients (2 days, IQR 1–3; *p* < 0.001).

Baseline characteristics stratified by PEP occurrence are presented in Table 1. Compared to patients without PEP, those who developed PEP were more likely to be female (73.68% vs. 54.01%, *p* = 0.002), have prior pancreatitis (19.74% vs. 8.73%, *p* = 0.007) or prior PEP (15.79% vs. 4.95%, *p* = 0.001), and present with asymptomatic choledocholithiasis (35.53% vs. 9.43%, *p* < 0.001). PEP patients also had higher stone burden, with greater median stone number (4 vs. 3, *p* = 0.004) and increased prevalence of multiple stones (56.58% vs. 42.45%, *p* = 0.031).

**Table 1:** Baseline characteristics stratified by post-ERCP pancreatitis occurrence

Variable	No PEP (*n* = 424)	PEP (*n* = 76)	*P*-value
Demographics			
Age (years), mean ± SD	62.26 ± 11.67	60.55 ± 10.49	0.235
Female sex, n (%)	229 (54.01)	56 (73.68)	0.002
BMI (kg/m²), mean ± SD	23.68 ± 3.19	24.04 ± 3.20	0.488
Medical History			
Prior pancreatitis, n (%)	37 (8.73)	15 (19.74)	0.007
Prior PEP, n (%)	21 (4.95)	12 (15.79)	0.001
Clinical Presentation			
Cholangitis, n (%)	207 (48.82)	17 (22.37)	< 0.001
Biliary colic, n (%)	177 (41.75)	32 (42.11)	
Asymptomatic, n (%)	40 (9.43)	27 (35.53)	
Laboratory Values			
Total bilirubin (mg/dL), median (IQR)	5.65 (3.09–11.17)	7.16 (2.89–11.46)	0.339
ALT (U/L), median (IQR)	66.54 (28.84-130.87)	69.03 (36.27-127.99)	0.965
AST (U/L), median (IQR)	54.71 (27.64–98.72)	46.77 (22.51–87.83)	0.221
WBC (×10⁹/L), median (IQR)	7.99 (5.93–10.13)	8.32 (7.11–10.18)	0.163
Imaging Findings			
CBD diameter (mm), median (IQR)	11.72 (9.66–13.69)	12.01 (8.50-14.13)	0.954
Dilated CBD, n (%)	363 (85.61)	61 (80.26)	0.306
Stone number, median (IQR)	3.00 (2.00–4.00)	4.00 (3.00–5.00)	0.004
Multiple stones (> 3), n (%)	180 (42.45)	43 (56.58)	0.031
Procedure Details			
Naive papilla, n (%)	290 (68.40)	59 (77.63)	0.139
Difficult cannulation, n (%)	101 (23.82)	31 (40.79)	0.003
Cannulation time (min), median (IQR)	3.66 (2.29–4.89)	4.43 (2.32–14.28)	0.112
PD instrumentation, n (%)	188 (44.34)	48 (63.16)	0.004
Precut sphincterotomy, n (%)	36 (8.49)	15 (19.74)	0.006
Pancreatic sphincterotomy, n (%)	36 (8.49)	11 (14.47)	0.152
Pancreatic stent placed, n (%)	86 (20.28)	10 (13.16)	0.196
Procedure time (min), median (IQR)	45.86 (34.10-57.73)	49.52 (38.29–60.18)	0.217

*Abbreviations: PEP* Post-ERCP pancreatitis,* SD* Standard deviation,* BMI* Body mass index* IQR* Interquartile range* ALT* Alanine aminotransferase, *AST* Aspartate aminotransferase,* WBC* White blood cell,* CBD* Common bile duct,* PD* Pancreatic duct

### Univariable analysis

Univariable logistic regression analysis (Table 2) identified multiple significant predictors of PEP. Patient-related factors significantly associated with PEP included female sex (OR 2.24, 95% CI 1.67–3.01), prior pancreatitis (OR 2.33, 95% CI 1.27–4.30), and prior PEP (OR 3.04, 95% CI 1.47–6.28). Asymptomatic choledocholithiasis demonstrated the strongest association among presentation types (OR 4.64, 95% CI 2.83–7.60) compared to biliary colic, while cholangitis was protective (OR 0.33, 95% CI 0.20–0.54).

**Table 2:** Univariable logistic regression analysis for post-ERCP pancreatitis

Variable	Odds Ratio	95% Confidence Interval	*P*-value
Age (per year increase)	0.99	0.97–1.01	0.235
Female sex	2.24	1.67–3.01	< 0.001
BMI (per kg/m² increase)	1.03	0.95–1.11	0.487
Prior pancreatitis	2.33	1.27–4.30	0.007
Prior PEP	3.04	1.47–6.28	0.003
Normal bilirubin	0.87	0.27–2.88	0.825
Dilated CBD	0.68	0.37–1.28	0.234
Multiple stones	1.71	1.22–2.39	0.002
Naive papilla	1.60	0.90–2.86	0.109
Difficult cannulation	2.1	1.40–3.15	< 0.001
PD instrumentation	2.05	1.49–2.82	< 0.001
Precut sphincterotomy	2.4	1.30–4.43	0.005
Pancreatic sphincterotomy	1.7	0.85–3.37	0.131
Pancreatic stent	0.63	0.33–1.20	0.16
Asymptomatic presentation	4.64	2.83–7.60	< 0.001
Cholangitis presentation	0.33	0.20–0.54	< 0.001

*Abbreviations: BMI* Body mass index,* PEP* Post-ERCP pancreatitis,* CBD* Common bile duct,* PD* Pancreatic duct

Procedure-related factors with significant associations included difficult cannulation (OR 2.10, 95% CI 1.40–3.15), pancreatic duct instrumentation (OR 2.05, 95% CI 1.49–2.82), and precut sphincterotomy (OR 2.40, 95% CI 1.30–4.43). Multiple stones demonstrated moderate association with PEP (OR 1.71, 95% CI 1.22–2.39). Naive papilla (OR 1.60, 95% CI 0.90–2.86, *p* = 0.109) and dilated CBD (OR 0.68, 95% CI 0.37–1.28, *p* = 0.234) did not reach statistical significance.

### Variable selection and final multivariable model

LASSO regression with 10-fold cross-validation selected 16 variables from the candidate predictor set, with optimal regularization parameter λ corresponding to maximum cross-validated AUC of 0.687. Applying our pre-specified reduction criteria (*p* < 0.05 in both univariable and multivariable analyses, aOR ≥ 1.5, clinical actionability, and EPV ≥ 10), we retained 5 predictors in the final model: prior PEP, asymptomatic presentation, difficult cannulation, pancreatic duct instrumentation, and precut sphincterotomy. This reduction ensured adequate EPV (76 events / 5 predictors = 15.2) to minimize overfitting while preserving clinically meaningful predictors with established biological rationale. prior PEP, asymptomatic presentation, difficult cannulation, pancreatic duct instrumentation, and precut sphincterotomy.

**Fig. 2:**
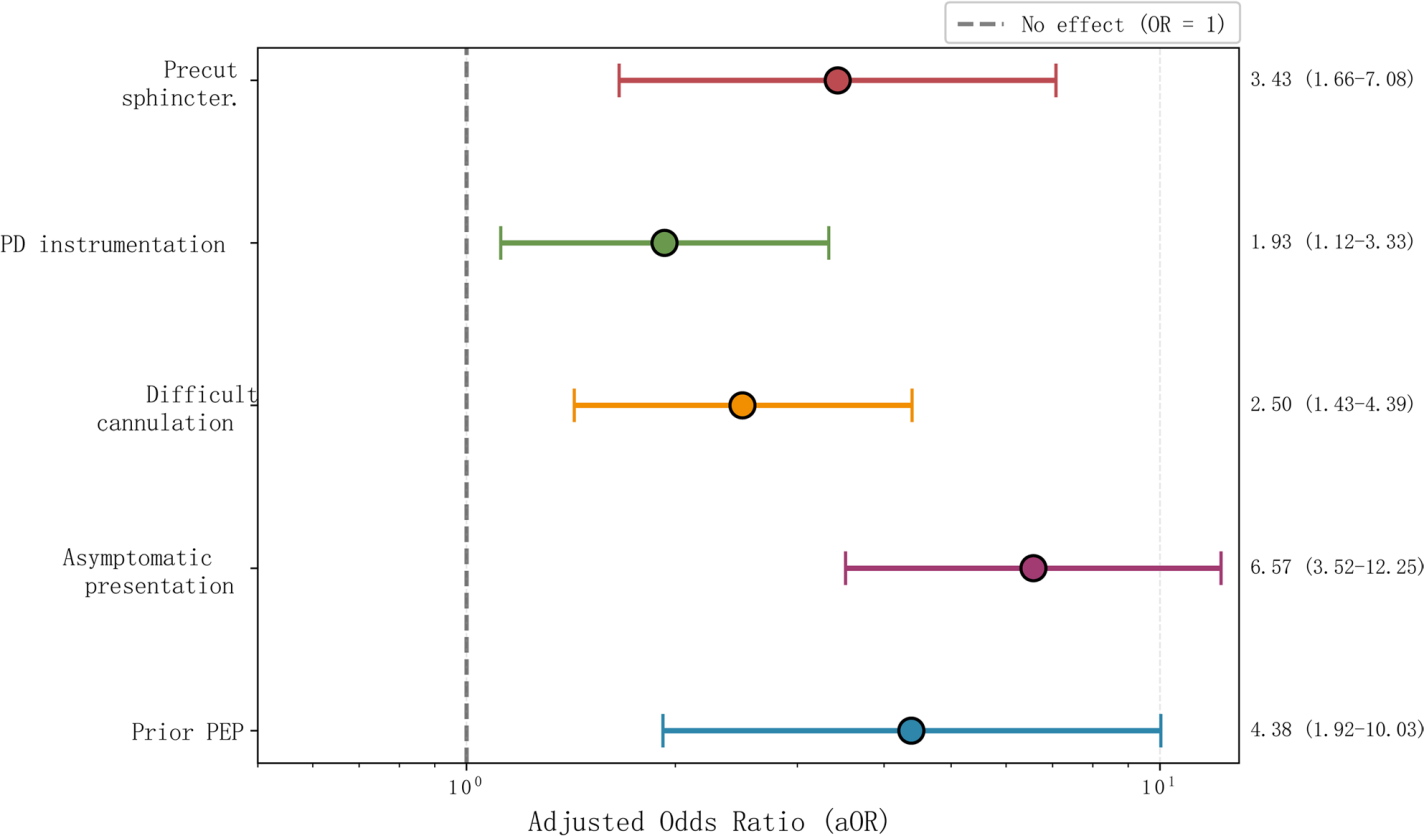
Forest plot showing adjusted odds ratios for independent predictors. Forest plot displaying adjusted odds ratios (aORs) with 95% confidence intervals for the five independent predictors in the final multivariable model. Asymptomatic choledocholithiasis demonstrated the strongest association (aOR 6.57, 95% CI 3.52–12.25), followed by prior post-ERCP pancreatitis (aOR 4.38, 95% CI 1.92–10.03), precut sphincterotomy (aOR 3.43, 95% CI 1.66–7.08), difficult cannulation (aOR 2.50, 95% CI 1.43–4.39), and pancreatic duct instrumentation (aOR 1.93, 95% CI 1.12–3.33). All predictors were statistically significant (*p* < 0.05)

The final multivariable logistic regression model (Table 3) demonstrated that all five predictors retained independent significance after adjustment. Prior PEP showed the strongest association (aOR 4.38, 95% CI 1.92–10.03, *p* < 0.001), followed closely by asymptomatic choledocholithiasis (aOR 6.57, 95% CI 3.52–12.25, *p* < 0.001). Difficult cannulation (aOR 2.50, 95% CI 1.43–4.39, *p* = 0.001), pancreatic duct instrumentation (aOR 1.93, 95% CI 1.12–3.33, *p* = 0.019), and precut sphincterotomy (aOR 3.43, 95% CI 1.66–7.08, *p* < 0.001) all demonstrated independent predictive value (Fig. 2).

**Table 3:** Final multivariable logistic regression model

Variable	Coefficient (β)	Standard Error	Adjusted Odds Ratio	95% Confidence Interval	*P*-value
Intercept	-3.0749	0.2818	0.05	0.03–0.08	< 0.001
Prior PEP	1.478	0.4222	4.38	1.92–10.03	< 0.001
Asymptomatic presentation	1.8823	0.3178	6.57	3.52–12.25	< 0.001
Difficult cannulation	0.9182	0.286	2.5	1.43–4.39	0.001
PD instrumentation	0.6568	0.2788	1.93	1.12–3.33	0.019
Precut sphincterotomy	1.232	0.37	3.43	1.66–7.08	< 0.001

*Abbreviations: PEP* Post-ERCP pancreatitis,* PD* Pancreatic duct

Full Model Equation: $$\mathrm{Logit}\left(\mathrm{PEP}\right)\:=-3.0749\:+1.4780\times\left(\mathrm{Prior}\:\mathrm{PEP}\right)\:+1.8823\times\left(\mathrm{Asymptomatic}\right)\:+0.9182\times$$$$\left(\mathrm{Difficult}\:\mathrm{cannulation}\right)\:+0.6568\times\left(\mathrm{PD}\:\mathrm{instrumentation}\right)\:+1.2320\times\left(\mathrm{Precut}\:\mathrm{sphincterotomy}\right)$$. Where all predictors are binary (0=absent, 1=present)

### Model performance

**Fig. 3:**
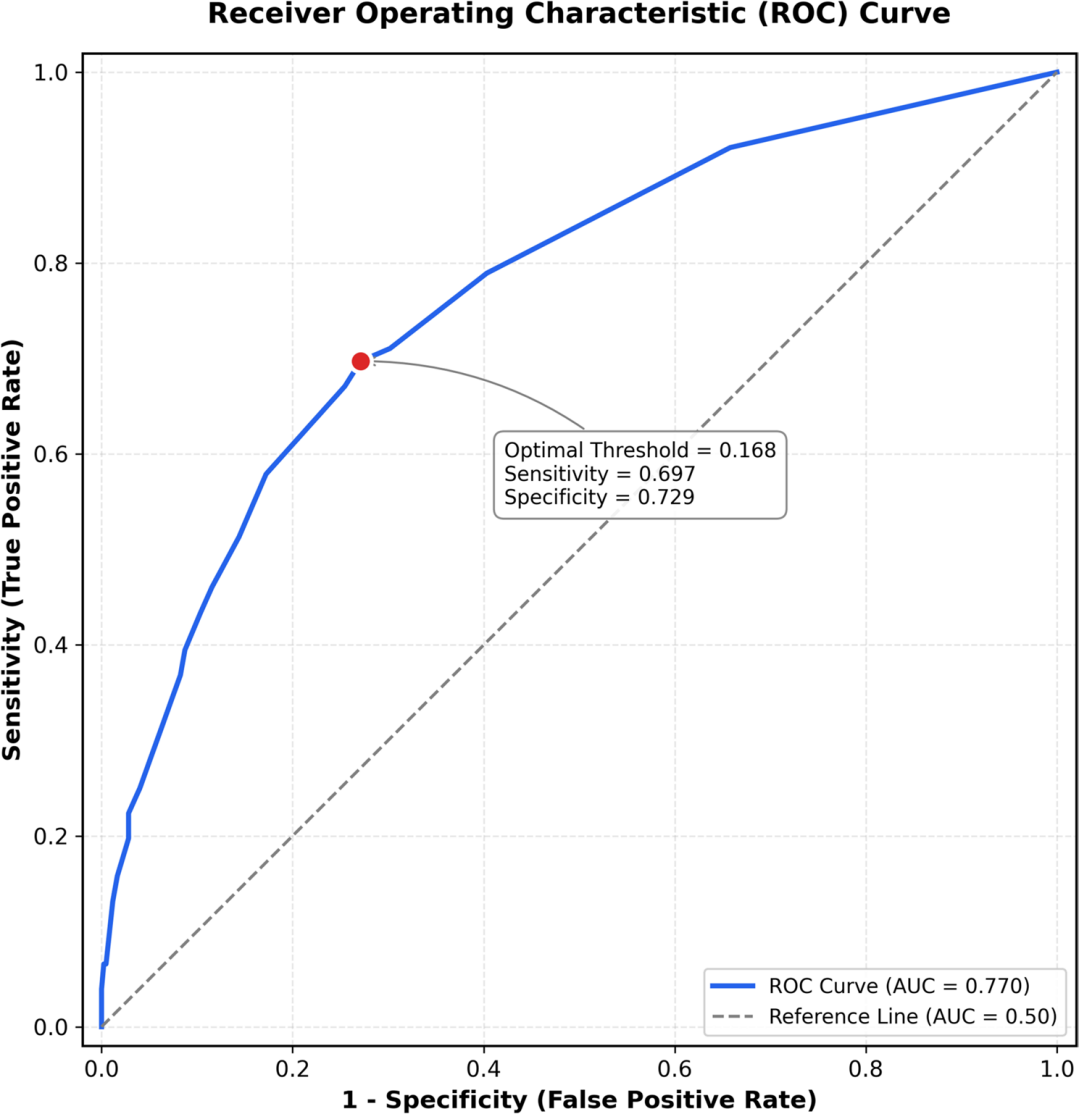
Receiver operating characteristic (ROC) curve for the final prediction model. The ROC curve demonstrates the model’s discrimination performance with an area under the curve (AUC) of 0.770. The optimal probability threshold of 0.168 (determined by Youden’s J statistic) achieved sensitivity of 0.697 and specificity of 0.729. The diagonal dashed line represents no discrimination (AUC = 0.50)

The model demonstrated excellent discrimination and calibration across both apparent and optimism-corrected metrics (Table 4). The apparent C-statistic was 0.770 (95% CI 0.748–0.792), with optimism-corrected C-statistic of 0.760 (95% CI 0.708–0.817) after bootstrap validation with 500 iterations, indicating acceptable discrimination (Fig. 3). At the optimal probability threshold of 0.168(determined by Youden’s J statistic), the model achieved sensitivity of 0.697, specificity of 0.729, PPV of 0.315, and NPV of 0.931.

**Table 4:** Model performance metrics

Metric	Apparent Performance	Optimism	Optimism-Corrected Performance
C-statistic	0.77	0.01	0.760 (95% CI 0.708–0.817)
Brier score	0.109	-0.004	0.113
Calibration slope	0.992	0.053	0.939
Calibration-in-the-large	0.001	-0.009	0.01

**Fig. 4:**
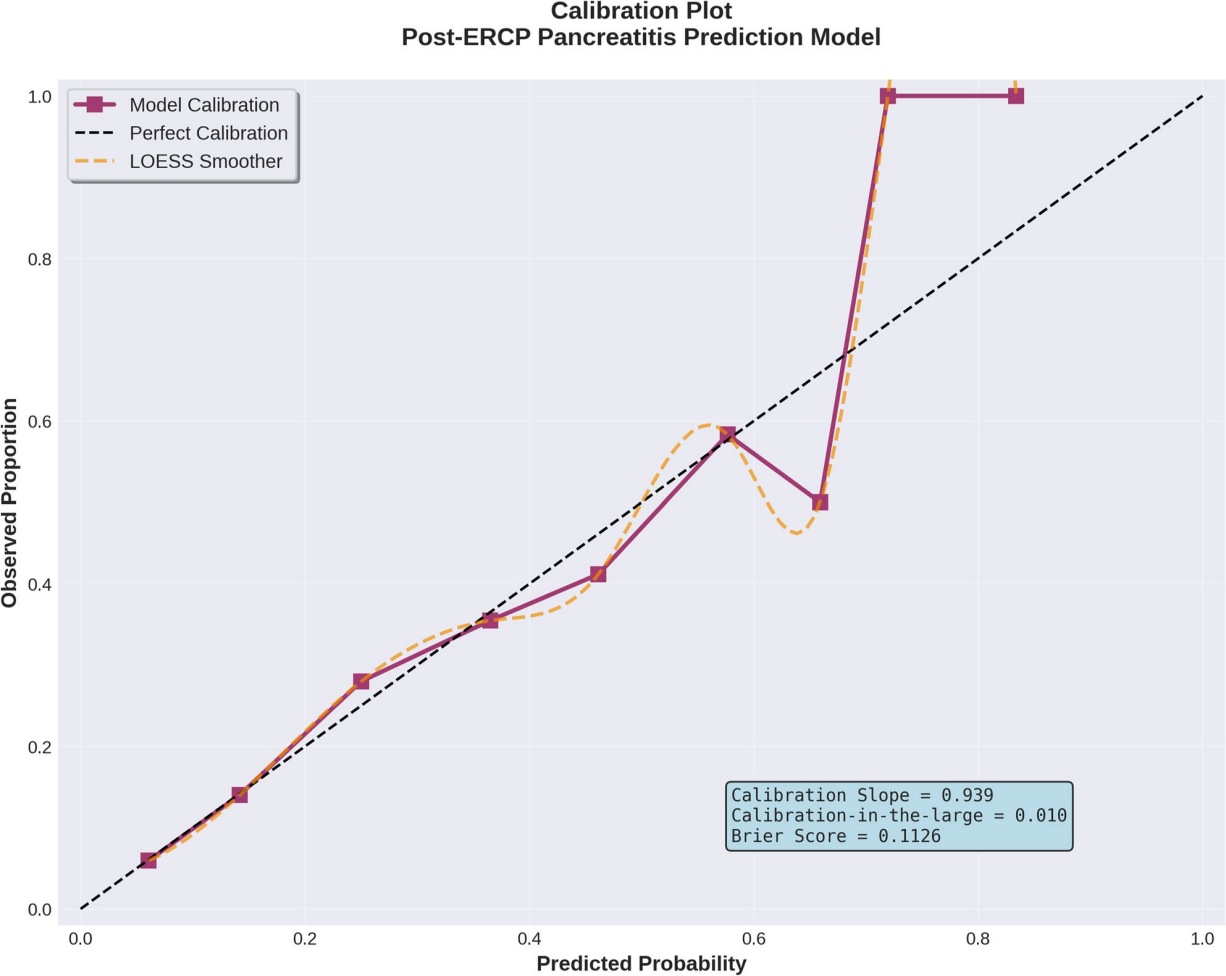
Calibration plot comparing predicted and observed post-ERCP pancreatitis rates. The calibration plot displays observed versus predicted event rates across deciles of predicted probability. The solid line represents LOESS smoothing of the observed data, while the dashed diagonal line indicates perfect calibration. The plot demonstrates excellent agreement between predicted and observed risks (Hosmer-Lemeshow χ²=1.862, *p* = 0.967), with minimal deviation from the ideal calibration line

The model demonstrated excellent calibration. The Hosmer-Lemeshow goodness-of-fit test yielded χ²=1.862 with 7 degrees of freedom (*p* = 0.967), indicating no evidence of poor fit. The optimism-corrected calibration slope of 0.939 (ideal = 1.0) suggested minimal overfitting, with observed versus predicted event rates showing strong agreement across the full risk spectrum (Fig. 4). The calibration-in-the-large value of 0.010 indicated that mean predicted probability closely matched the observed event rate.

### Risk score development and stratification

The regression coefficients were transformed into an integer-based risk score by scaling to the smallest coefficient (pancreatic duct instrumentation, β = 0.657) and rounding (Table 5). The resulting point system assigned: prior PEP (2 points), asymptomatic choledocholithiasis (3 points), difficult cannulation (1 point), pancreatic duct instrumentation (1 point), and precut sphincterotomy (2 points), with total scores ranging from 0 to 9 points.

**Table 5:** Simplified risk score system for clinical application

Risk Factor	Points
Prior post-ERCP pancreatitis	2
Asymptomatic choledocholithiasis	3
Difficult cannulation (> 5 min or > 5 attempts)	1
Pancreatic duct instrumentation	1
Precut sphincterotomy	2
Total Possible Score	0–9

Patients were categorized into three risk groups based on observed PEP rates: low risk (0–1 points), moderate risk (2–3 points), and high risk (≥ 4 points). This stratification achieved meaningful separation with observed PEP rates of 6.92% (22/318) in the low-risk group, 21.85% (26/119) in the moderate-risk group, and 44.44% (28/63) in the high-risk group (Table 6 and Fig. 4). The risk gradient demonstrated approximately 3.2-fold increase from low to moderate risk, and 6.4-fold increase from low to high risk.

**Table 6:** Risk stratification performance

Risk Category	Score Range	*N* Patients (%)	PEP Cases	Observed PEP Rate (%)	Mean Predicted Probability (%)
Low Risk	0–1	318 (63.60)	22	6.92	7.84
Moderate Risk	2–3	119 (23.80)	26	21.85	19.67
High Risk	≥ 4	63 (12.60)	28	44.44	41.29

*Abbreviations: PEP P*ost-ERCP pancreatitis

Distribution analysis revealed that 318 patients (63.60%) were classified as low risk, 119 (23.80%) as moderate risk, and 63 (12.60%) as high risk. The majority of PEP cases (71.05%, 54/76) occurred in the moderate and high-risk categories, while only 28.95% (22/76) occurred in low-risk patients, demonstrating effective risk concentration.

## Discussion

In this single-center retrospective cohort study of 500 consecutive patients undergoing therapeutic ERCP for choledocholithiasis, we developed and internally validated a parsimonious five-predictor model demonstrating acceptable discrimination (bootstrap optimism-corrected C-statistic 0.760, 95% CI 0.708–0.817) and satisfactory calibration for predicting post-ERCP pancreatitis. The derived integer-based risk score stratified patients into clinically meaningful risk categories, with PEP rates ranging from 6.92% in low-risk to 44.44% in high-risk groups—a 6.4-fold gradient facilitating individualized prophylaxis decisions. To our knowledge, this represents one of the few choledocholithiasis-specific prediction models rigorously developed using contemporary methodological standards, with particular strengths including LASSO-based variable selection, comprehensive bootstrap internal validation, and transparent reporting following TRIPOD guidelines.

### Comparison with existing prediction models

Our model’s discrimination performance (C-statistic 0.760) aligns favorably with the range reported for previously published PEP prediction models. A recent systematic review of 24 prediction models found C-statistics ranging from 0.70 to 0.92, with median values around 0.75–0.80.8 Among choledocholithiasis-specific models, Yan et al. [4] reported exceptional discrimination (C-statistic 0.915 in derivation, 0.838 in external validation) in a Chinese cohort of non-elderly CBD stone patients, though their model included 8 variables and employed machine learning approaches that may limit interpretability. Fujita et al. [12] developed a practical 7-factor scoring system achieving C-statistic of 0.799 with external validation in a Japanese population, incorporating both patient and procedural factors.

Several recent machine learning-based approaches have demonstrated competitive or superior discrimination. Takahashi et al. [36] reported random forest models with AUROC 0.821 in development and 0.770 in external validation, while Brenner et al. [37] achieved C-statistics of 0.70–0.74 using gradient-boosted machines trained on 12 randomized trials. However, black-box machine learning models often sacrifice interpretability for modest performance gains and may be less suitable for point-of-care decision-making without embedded implementation in electronic health record systems [38]. 

Importantly, our model demonstrated superior calibration compared to most published models. The Hosmer-Lemeshow p-value of 0.967, calibration slope near 1.0 (0.939), and calibration-in-the-large near 0 (0.010) collectively indicate excellent agreement between predicted and observed risks across the probability spectrum—a critical but often overlooked aspect of prediction model performance [29]. Many existing models report only discrimination metrics, with systematic reviews revealing that 62% of PEP prediction studies fail to assess calibration adequately [9]. Poor calibration undermines clinical utility even when discrimination is acceptable, as miscalibrated predictions may lead to inappropriate resource allocation and suboptimal shared decision-making.

### Clinical implications and practical application

The derived risk score offers several advantages for clinical implementation. First, it requires only five readily available variables, with two (prior PEP and asymptomatic presentation) knowable pre-procedure and three (difficult cannulation, pancreatic duct instrumentation, and precut sphincterotomy) ascertainable intra-procedurally. This temporal split enables both pre-procedure risk counseling and dynamic intra-procedure prophylaxis decisions. Second, the integer-based scoring system (0–9 points) is easily calculable without computational aids, facilitating bedside use. Third, the three-tiered risk stratification provides actionable thresholds aligned with current prophylaxis options.

Under current ASGE guidelines [13], rectal NSAIDs are recommended for all patients regardless of risk status, based on robust evidence from multiple meta-analyses demonstrating relative risk reduction of approximately 35–40% [39]. Our low-risk category (baseline PEP rate 6.92%) would be expected to achieve post-prophylaxis rates around 4–5% with NSAIDs alone, potentially obviating the need for additional interventions. Conversely, high-risk patients (baseline rate 44.44%) may warrant aggressive combination prophylaxis despite recent trial results questioning additive benefits of pancreatic stenting beyond NSAIDs [14]. The moderate-risk group (21.85%) represents the greatest decision-making challenge and may benefit most from individualized approaches considering patient preferences, local expertise, and resource availability. It should be emphasized that these proposed clinical applications remain hypothetical and were not directly tested in this study. Our model provides risk estimates, not treatment recommendations. Whether implementing risk-stratified prophylaxis based on our score improves patient outcomes compared to current practice requires prospective evaluation in randomized or pragmatic trials.

It is noteworthy that our observed overall PEP rate (15.20%) substantially exceeds the commonly cited range of 3–10% in general ERCP populations [2]. However, our rate aligns with recent meta-analyses reporting 14.1% incidence in high-risk groups [2] and with choledocholithiasis-specific studies from Asian populations reporting 8–15% rates [4, 6]. The elevated incidence likely reflects several factors: (1) enrichment for high-risk features (13.4% asymptomatic presentation, 68% naive papilla); (2) single-center tertiary referral patterns concentrating complex cases; (3) sensitive outcome ascertainment with routine 24-hour enzyme monitoring; and (4) potential geographic/ethnic variations in PEP susceptibility reported in Asian cohorts [9]. 

### Key risk factor insights

Our findings reinforce and extend several important observations regarding PEP risk factors in choledocholithiasis patients. Asymptomatic presentation emerged as one of the strongest predictors (aOR 6.57), corroborating mounting evidence that incidentally discovered CBD stones confer paradoxically elevated risk compared to symptomatic disease [3, 4, 6]. A recent meta-analysis reported pooled OR 4.76 for asymptomatic versus symptomatic choledocholithiasis [7], though definitions varied across studies. Mechanistically, this counterintuitive finding may reflect less inflamed, more capacious bile ducts in asymptomatic patients, potentially facilitating deeper pancreatic duct cannulation and manipulation. Alternatively, symptomatic presentations (particularly cholangitis) may elicit protective physiological responses or necessitate procedural modifications that coincidentally reduce PEP risk.

Prior PEP history (aOR 4.38) and general pancreatitis history represent well-established risk factors, likely reflecting underlying pancreatic susceptibility—whether anatomic (small-diameter pancreatic duct, long common channel), genetic (CFTR mutations, PRSS1 variants), or related to recurrent subclinical pancreatic injury [5, 7]. Difficult cannulation and precut sphincterotomy, procedure-related factors partially under endoscopist control, emphasize opportunities for technique refinement and early recognition of challenging cases warranting prophylactic interventions before proceeding with repeated attempts.

Notably, several factors commonly featured in other prediction models did not emerge as independent predictors in our analysis. Female sex and young age, frequently cited patient-related risk factors [5, 7], showed only marginal associations in multivariable analysis after accounting for other predictors. This may reflect collinearity with sphincter of Oddi function and presentation patterns, or may indicate that these demographic factors serve as proxies for more direct mechanistic determinants captured by our procedural variables.

### Methodological strengths and innovations

This study adheres to rigorous contemporary standards for clinical prediction model development, incorporating several methodological strengths. First, we employed LASSO regularization for variable selection, avoiding pitfalls of traditional stepwise selection (inflation of coefficients, biased standard errors, excessive optimism) [24, 40]. Second, we performed comprehensive bootstrap internal validation with 500 iterations calculating optimism correction for all performance metrics, providing realistic estimates of model transportability [8, 25, 26]. Third, we assessed both discrimination and calibration using multiple complementary measures, recognizing that calibration represents the “Achilles heel” of predictive analytics yet is critical for clinical application.30 Fourth, we transparently reported the full model equation enabling external validation and pre-specified our modeling strategy before data access following TRIPOD recommendations [16, 17, 41]. 

Our approach to risk score derivation, converting regression coefficients to integer points while preserving rank order of effect sizes, balances statistical rigor with clinical practicality [34, 35]. Alternative approaches such as recursive partitioning (classification trees) sacrifice statistical efficiency, while maintaining continuous predicted probabilities enhances discrimination at the cost of implementation barriers. Our three-tiered stratification scheme represents a pragmatic middle ground, providing sufficient granularity for clinical decision-making without unnecessary complexity.

### Limitations and future directions

Several limitations warrant consideration. Most critically, this study represents single-center retrospective experience with internal validation only; external validation in independent, geographically diverse cohorts is essential before any clinical application can be recommended. The retrospective design and 6-year study period may introduce temporal heterogeneity from practice evolution. Additionally, three of five predictors (difficult cannulation, pancreatic duct instrumentation, precut sphincterotomy) are only ascertainable intra-procedurally, limiting pre-procedure risk counseling to two variables (prior PEP and clinical presentation). This temporal constraint means complete risk stratification is only possible after the procedure has commenced. Calibration, in particular, often deteriorates substantially in external populations due to case-mix differences and outcome prevalence shifts [42, 43]. Third, our sample size, while adequate for developing a parsimonious model, precluded exploration of complex interactions or non-linear relationships that might enhance predictive accuracy. Fourth, unmeasured confounders inherent to observational designs (prophylactic medication dosing variations, individual endoscopist technique and experience, subtle differences in balloon dilation protocols) may bias effect estimates.

Fifth, procedure-related predictors (difficult cannulation, pancreatic duct instrumentation) are only ascertainable intra-procedurally, limiting pre-procedure risk counseling to three predictors (prior PEP, presentation, papilla status). Future models incorporating preprocedural imaging features (pancreatic duct diameter, ampullary morphology) or novel biomarkers might improve prospective risk stratification. Sixth, we did not assess the model’s impact on clinical decision-making or patient outcomes. Implementation studies evaluating whether model-guided prophylaxis improves PEP prevention, reduces unnecessary interventions, or enhances cost-effectiveness represent critical next steps [38, 44]. 

Seventh, our cohort consisted predominantly of Han Chinese patients, and genetic, anatomic, or environmental factors may limit generalizability to other populations. Eighth, we did not systematically collect data on prophylactic interventions. At our institution during the study period, rectal indomethacin (100 mg) was routinely administered, prophylactic pancreatic stent placement was performed at the endoscopist’s discretion (approximately 19% of cases as shown in Table 1), and aggressive hydration protocols were not uniformly implemented. This incomplete systematic capture of prophylaxis measures—which are often applied based on perceived risk—may influence observed PEP rates and model calibration. The transportability of our model to centers with different prophylaxis practices (such as universal pancreatic stenting or aggressive hydration protocols) requires careful consideration, as baseline PEP rates and risk factor associations may vary accordingly. Finally, the retrospective design precluded standardized collection of certain potentially relevant variables (sphincter of Oddi manometry findings, minor papilla anatomy, detailed balloon dilation parameters including duration and pressure) that might refine risk prediction.

Future research should prioritize external validation in multicenter prospective cohorts, ideally from diverse geographic regions and practice settings. Incorporation of novel predictors such as artificial intelligence-based image analysis of cholangiograms [45], real-time papillary morphology assessment, or circulating biomarkers (genetic polymorphisms, baseline serum trypsinogen) may enhance model performance. Development of dynamic prediction models that update risk estimates intra-procedurally as additional information accrues (cumulative pancreatic duct contrast injection volume, number of deep pancreatic duct wire passages, wire trauma score) represents an intriguing avenue. Integration with electronic health records through automated risk calculators with clinical decision support could facilitate implementation while enabling continuous model recalibration and quality improvement monitoring [38]. 

Ultimately, demonstrating that risk-stratified prophylaxis informed by our model improves patient outcomes compared to current practice will be essential for clinical adoption. Pragmatic trials randomizing high-risk patients (≥ 4 points) to intensified prophylaxis bundles (combination NSAID + pancreatic stent + aggressive hydration) versus standard care could establish clinical utility beyond predictive accuracy. Simultaneously, trials evaluating whether low-risk patients (< 2 points) can safely forego pancreatic stenting even in settings where it might otherwise be considered could improve resource allocation and reduce complications associated with stent placement itself.

## Conclusions

We developed and internally validated a practical, choledocholithiasis-specific risk score demonstrating robust discrimination and excellent calibration for predicting post-ERCP pancreatitis. The five-predictor model incorporates readily available clinical variables and stratifies patients into low (6.92%), moderate (21.85%), and high (44.44%) risk categories, providing a framework for individualized risk assessment that may inform clinical decision-making pending external validation. Adherence to contemporary methodological standards including LASSO variable selection, bootstrap internal validation, and comprehensive performance assessment strengthens confidence in the model’s validity. External validation in independent, multicenter cohorts and implementation studies evaluating clinical impact represent critical next steps toward improving risk stratification and prevention strategies for this common and consequential ERCP complication.

## Data Availability

De-identified individual participant data and statistical code will be made available upon reasonable request to the corresponding author following publication, subject to institutional approval and data sharing agreement.

## Abbreviations

ERCP: Endoscopic Retrograde Cholangiopancreatography
PEP: Post-ERCP Pancreatitis
LASSO: Least Absolute Shrinkage and Selection Operator
aOR: Adjusted Odds Ratio
CI: Confidence Interval
CT: Computed Tomography
MRCP: Magnetic Resonance Cholangiopancreatography
ASGE: American Society for Gastrointestinal Endoscopy
NSAIDs: Nonsteroidal Anti-Inflammatory Drugs
TRIPOD: Transparent Reporting of a multivariable prediction model for Individual Prognosis or Diagnosis
STROBE: Strengthening the Reporting of Observational Studies in Epidemiology
BMI: Body Mass Index
ALT: Alanine Aminotransferase
AST: Aspartate Aminotransferase
ALP: Alkaline Phosphatase
GGT: Gamma-Glutamyl Transferase
CBD: Common Bile Duct
AUC: Area Under the Curve
ROC: Receiver Operating Characteristic
PPV: Positive Predictive Value
NPV: Negative Predictive Value
SD: Standard Deviation
IQR: Interquartile Range
EPV: Events Per Variable
WBC: White Blood Cell
PD: Pancreatic Duct
LOESS: Locally Estimated Scatterplot Smoothing
CFTR: Cystic Fibrosis Transmembrane Conductance Regulator
PRSS1: Protease Serine 1
ESGE: European Society of Gastrointestinal Endoscopy
JGH: Journal of Gastroenterology and Hepatology
AUROC: Area Under the Receiver Operating Characteristic Curve
AI: Artificial Intelligence
NASSS: Nonadoption, Abandonment, Scale-up, Spread, and Sustainability Framework
